# UK Dementia Incidence Doesn't Vary across Sites with Known Variation in Vascular Risk

**DOI:** 10.1371/journal.pmed.0020275

**Published:** 2005-08-23

Dementia remains an incurable condition and its increasing prevalence is a deeply worrying aspect of the “graying” of the population. An important question for researchers is to establish whether dementia incidence, prevalence, and national history vary from one location to another. Incidence studies are particularly valuable for less biased comparison of disease occurrence, as well as being essential for policy makers. Many biases can, however, be introduced in such studies. Dropout and mortality are particular reasons for concern.

Most of the numerous studies of dementia incidence have been restricted to single sites. Authors have frequently attempted to assess whether rates in a given study are similar to those obtained elsewhere. However, variations between studies in the methodology employed make such comparisons unreliable. Where within-country variations in incidence have been noted, as has happened in the US, they have often been ascribed to methodological differences, but one cannot be certain whether this is the case.

Risk factors for other chronic disorders common in old age (notably cardiovascular disease and cancers) do vary in their prevalence between and within countries. In the UK, for example, the incidence of stroke is known to vary considerably across the country. A high proportion of dementia patients are thought to have a vascular component to their dementia, and it has been assumed that dementia incidence could be reduced if vascular risk were better controlled. One way to test this hypothesis is to compare sites with known variation in vascular risk to assess whether there is also variation in the incidence of dementia.

The Medical Research Council Cognitive Function and Ageing Study (MRC CFAS) is a multi-site, population-based study in the UK of individuals aged 65 years and over living in the community, including institutions. Diverse sites have been chosen, with varying exposures of potential importance in dementia. A two-phase two-wave design has been employed, with the waves two years apart. A standard set of instruments for the diagnosis of dementia is used throughout. CFAS now publishes incidence estimates from five sites, using likelihood-based methods to compare the first two waves of interviews.

Predictably, incidence rates of dementia, for both sexes, were found to rise with age, from 6.7 per 1,000 person years at age 65–69 years to 68.5 per 1,000 person years at age 85 years and above. The authors estimate that around 163,000 new cases of dementia occur in England and Wales each year. However, there was no convincing evidence of variation across sites, and the incidence rates do not reflect the variations in the prevalence of possible risk factors in these sites. We therefore cannot assume that action to reduce vascular risk will have a significant impact on dementia incidence.

Another issue addressed by the study is previous suggestions in the literature that dementia incidence rates might be lower in the oldest age groups. The limited number of respondents in these age groups in previous studies made it impossible to test this hypothesis. The CFAS, however, found no evidence of any such tailing off in incidence, which also has implications for policy and planning.

The CFAS is important because it provides the first multi-site comparison of incidence rates in ethnically homogeneous populations within a country, and within Europe, using identical methodology across sites. The methodological approach developed for the study will also be of value for researchers undertaking other studies of dementia incidence, and in other chronic disease studies involving a two-phase selection process.

